# Combination of single-cell and bulk RNA seq reveals the immune infiltration landscape and targeted therapeutic drugs in spinal cord injury

**DOI:** 10.3389/fimmu.2023.1068359

**Published:** 2023-01-19

**Authors:** Qing Zhang, Beibei Yu, Yongfeng Zhang, Yunze Tian, Shijie Yang, Yongfeng Chen, Haining Wu

**Affiliations:** ^1^ Key laboratory of Shaanxi Province for Craniofacial Precision Medicine Research, College of Stomatology, Xi’an Jiaotong University, Xi’an, China; ^2^ Department of Neurourgery, the Second Affiliated Hospital of Xi’an Jiao Tong University, Xi’an, China; ^3^ Department of Orthopaedics, Xijing Hospital, Fourth Military Medical University, Xi’an, China; ^4^ State Key Laboratory of Cancer Biology, Department of Biochemistry and Molecular Biology, Fourth Military Medical University, Xi’an, China

**Keywords:** spinal cord injury, immune infiltration, macrophages/microglia, decitabine, ScRNA-seq

## Abstract

**Background:**

In secondary spinal cord injury (SCI), the immune microenvironment of the injured spinal cord plays an important role in spinal regeneration. Among the immune microenvironment components, macrophages/microglia play a dual role of pro-inflammation and anti-inflammation in the subacute stage of SCI. Therefore, discovering the immune hub genes and targeted therapeutic drugs of macrophages/microglia after SCI has crucial implications in neuroregeneration. This study aimed to identify immune hub genes and targeted therapeutic drugs for the subacute phase of SCI.

**Methods:**

Bulk RNA sequencing (bulk-RNA seq) datasets (GSE5296 and GSE47681) and single-cell RNA sequencing (scRNA-seq) dataset (GSE189070) were obtained from the Gene Expression Omnibus database. In the bulk RNA-seq, the R package ‘limma,’ ‘WGCNA,’ and ‘CIBERSORT’ were used to jointly screen key immune genes. Subsequently, the R package ‘Seurat’ and the R package ‘celldex’ were used to divide and annotate the cell clusters, respectively. After using the Autodock software to dock immune hub genes and drugs that may be combined, the effectiveness of the drug was verified using an *in vivo* experiment with the T9 SCI mouse model.

**Results:**

In the bulk-RNA seq, *B2m*, *Itgb5*, and *Vav1* were identified as immune hub genes. Ten cell clusters were identified in scRNA-seq, and *B2m and Itgb5* were mainly located in the microglia, while *Vav1* was mainly located in macrophages. Molecular docking results showed that the proteins corresponding to these immune genes could accurately bind to decitabine. In decitabine-treated mice, the pro-inflammatory factor (TNF-α, IL-1β) levels were decreased while anti-inflammatory factor (IL-4, IL-10) levels were increased at 2 weeks post-SCI, and macrophages/microglia transformed from M1 to M2. At 6 weeks post-SCI, the neurological function score and electromyography of the decitabine treatment group were also improved.

**Conclusion:**

In the subacute phase of SCI, *B2m, Itgb5, and Vav1* in macrophages/microglia may be key therapeutic targets to promote nerve regeneration. In addition, low-dose decitabine may promote spinal cord regeneration by regulating the polarization state of macrophages/microglia.

## 1 Introduction

Spinal cord injury (SCI) is commonly caused by external physical impact (e.g., traffic accidents, violence, or sports-related injury), and it has devastating physical and social consequences for patients and their families. The high complexity of the spinal cord and imbalanced inflammatory microenvironment following SCI hinder neural regeneration and functional recovery. SCI patients have multiple disabilities in motosensory function (1). However, despite its associated severe neurological dysfunction and poor prognosis, the clinical treatment for SCI mainly involves palliative surgical decompression and glucocorticoids (2, 3) These strategies fail to prevent inflammatory injury and neurodegeneration in the injured spinal cord. Thus, improved treatment modalities are a critical unmet need in SCI management.

SCI can be divided into three phases, namely, acute (<48 hours), subacute(>48 hours, <2 weeks), and intermediate and chronic phases (>2 weeks, <6 months) (4). In the subacute phase, neuronal loss and axon and myelin necrosis activate macrophages to release proinflammatory cytokines, which lead to an extensive inflammatory response in the SCI microenvironment (5, 6). Subsequently, the blood–spinal cord barrier is destroyed, resulting in immune microenvironment disorder and poor regeneration of the injured spinal cord (7). Macrophages/microglia plays an important role in maintaining the immune homeostasis of the CNS microenvironment. Homeostatic microglia could promote neuronal development, axonal regeneration and pruning of neuronal/synaptic side branches, and once it was activated or in the microenvironment of disease, microglia are transformed into disease-associated microglia (DAM) (8). In spinal cord injury, Milich et al. classified DAM as inflammatory, dividing, and migrating microglia based on the single-cell RNA sequencing (scRNA-seq) and flow cytometry. However, overall, macrophages/microglia play dual roles in the pathogenesis and prognosis of SCI, that is, anti-inflammatory and anti-inflammatory effects (9). As such, a deeper understanding of the heterogeneity of the immune microenvironment after SCI and targeting the macrophages/microglia may be helpful for establishing the optimal regeneration strategy after SCI.

RNA-seq high-throughput sequencing is currently widely used in biology owing to its convenience for discovering immune hub genes and distinguishing the heterogeneity of immune cells. This study aimed to identify immune hub genes and targeted therapeutic drugs for the subacute phase of SCI. Towards this goal, we screened the key immune genes in the subacute stage post-SCI through differential gene expression analysis, weighted gene coexpression network analysis (WGCNA), and immune infiltration analysis in the bulk RNA sequencing (bulk RNA-seq) datasets GSE5296 and GSE47681. Subsequently, in the scRNA-seq dataset GSE189070, the composition of cell clusters and cell clusters where these immune genes were located were identified. Finally, based on the key immune genes, we identified the drug that may promote the regeneration of SCI mice by switching the polarization state of macrophages/microglia and verified its effectiveness through *in vivo* experiments.

## 2 Materials and methods

### 2.1 Microarray datasets

**Figure 1:**
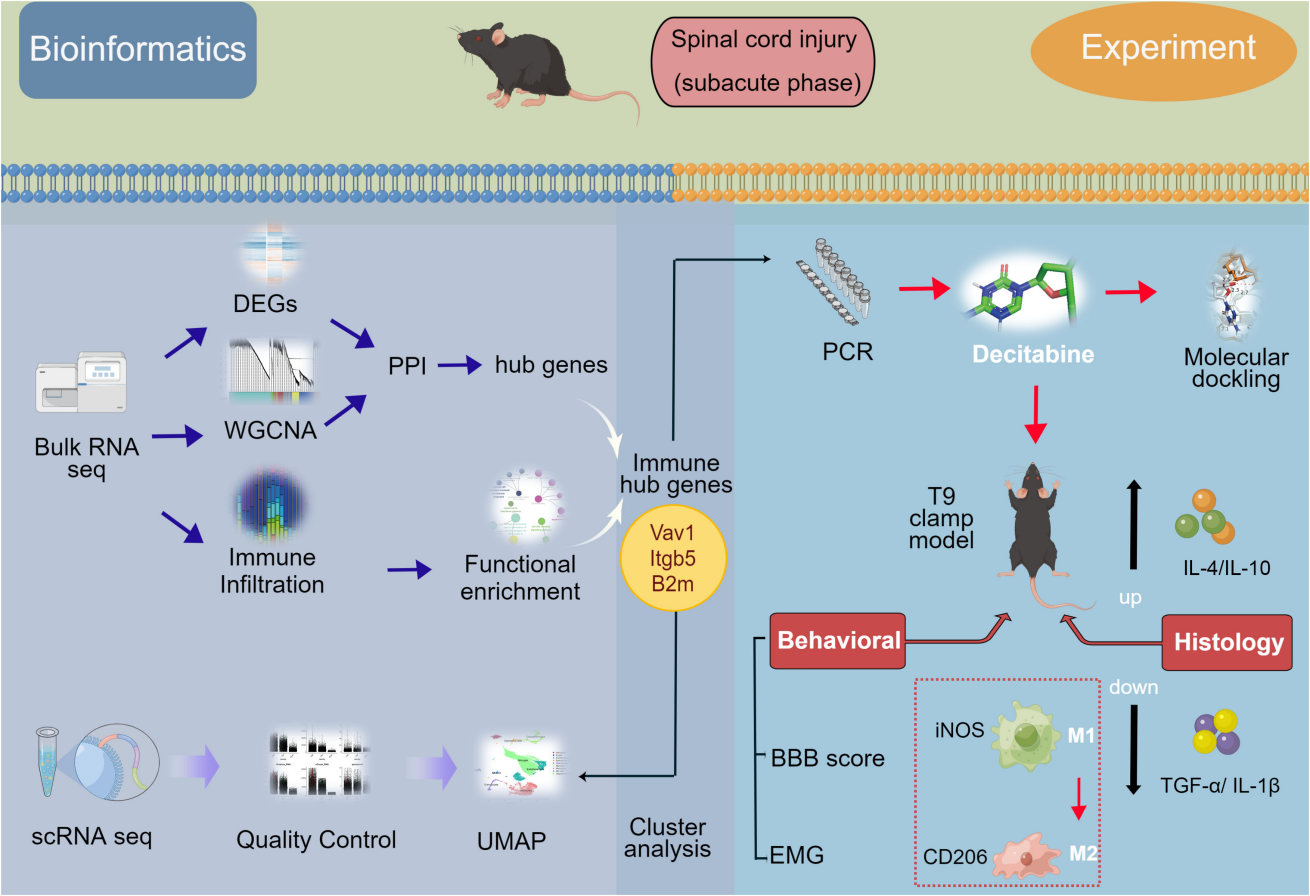
Flow-diagram for the study. SCI, spinal cord injury; DEGs, differentially expressed genes; PPI, protein-protein interaction; WGCNA, weighted gene coexpression network analysis (WGCNA); UMAP, uniform manifold approximation and projection; BBB score, Basso-Beattie-Bresnahan locomotion testing score; EMG, electromyography.

Open-access microarray data were retrieved from the Gene Expression Omnibus database^1^ at the National Center for Biotechnology Information. Three datasets, namely, GSE5296, GSE47681, and GSE189070, were selected according to the following inclusion criteria: (1) samples of the experimental group were collected from mice after SCI in the thoracic segment and without drug or other interventions and (2) microarray of the SCI sample was sequenced in the subacute phase (2–14 days). The bulk RNA-seq datasets (GSE5296 and GSE47681), all based on the GPL1261 platform and contained 10 samples (four control and six SCI) and 12 samples (four control and eight SCI) were sequenced at 3 days post-injury (dpi) and 7 dpi, respectively (10, 11). The GSE189070 dataset based on the GPL24247 platform containing single-cell RNA data from six mice in the uninjured group and 12 mice in the injured group were sequenced at 3 dpi and 7 dpi, which corresponds to the sequencing time of bulk RNA seq datasets (12). The study flow chart is shown in **Figure 1**.

### 2.2 Data processing and screening of key regulatory genes

R version 4.1.0 (R Foundation for Statistical Computing, Vienna, Austria) was used for all statistical analyses. Using the ‘combat’ function (in the ‘sva’ package), batch effects were removed after the combination of the bulk RNA-seq datasets (GSE5296 and GSE47681). To visualize the separation of the uninjured and injured group, principal component analysis was performed using the ‘factoextra’ package. Under the filter of |log 2 (fold-change) (FC) | >1 and an adjusted p<0.05, differentially expressed genes (DEGs) were identified using the bioconductor package “limma.”

WGCNA, which can identify gene modules with similar expression patterns, map regulatory networks between gene sets, and identify key regulatory genes (13), was used to perform module analysis. After selecting genes with variance greater than 25%, the power of 8 was determined by pickSoftThreshold function. Through average linkage hierarchical clustering, the genes with similar expression profiles were categorized to gene modules using the TOM-based dissimilarity measure. Ultimately, the correlation between each module and the sample trait (presence or absence of SCI) was assessed. Modules with the highest correlation were used to intersect with DEGs using the ‘VennDiagram’ package.

### 2.3 Analysis of key regulatory genes and immune infiltration

The protein–protein interaction network analysis was carried out by using Metascape and STRING database^2^ ^,3^. The most significant modules were identified using the MCODE plug of Cytoscape, with a cutoff score of 5. Gene Ontology and Kyoto Encyclopedia of Genes and Genomes enrichment analysis was performed by the ClueGO plug of Cytoscape software, with P<0.05 and gene counts ≥ 3 set as the cut-off criteria for enrichment items.

For immune infiltration analysis, CIBERSORT algorithm^4^ was applied to compute immune cell abundance of the samples. The ‘pheatmap’ package was used to visualize the content of 22 kinds of immune cells in spinal cord tissues. Spearman’s correlation analysis was then performed to detect the correlation coefficients between immune cells and gene expression levels. The criteria for selection of key immune genes related to SCI were as follows: >2 immune cell correlation, correlation coefficient >0.3, and p<0.05.

### 2.4 ScRNA-seq data processing and identification of cell types

For single-cell genomics analysis, the processed data were analyzed using the ‘Seurat’ package. For downstream analyses, we excluded cells with a total number of expressed genes exceeding 6,000, mitochondrial gene counts exceeding 15%, and genes expressed in more than three cells. Subsequently, the expression matrix was standardized using the log2 (CPM+1) values as input matrix. High variable genes were identified using the ‘FindVariableGenes’ function, and principal component analysis was performed. The dimension reduction method for single-cell visualization was uniform manifold approximation and projection (UMAP). Then, differential expression analysis was performed using ‘FindAllMarkers’ function with |log 2 (fold-change) |>0.3 and adjusted p<0.05. ‘DimPlot’ function was used to visualize the single-cell plot, and ‘FeaturePlot’ function was used to visualize the gene expression plot. Thereafter, the single-cell subgroups were annotated using the ‘MouseRNAseqData’ function in the ‘celldex’ package. Finally, the cellular crosstalk was calculated with “CellChat” package.

### 2.5 Molecular modeling analysis

We hypothesized that the FDA-approved drugs and experimental compounds may be combined with key immune targets using the DSigDB database^5^. The two-dimensional structures of selected compounds were downloaded from the Pubchem database^6^. The RCSB Protein Data Bank^7^ was used to obtain the structures of the proteins. We specified three PDBQT files: proteins rigid, flexible, and ligands. The AutoDock 4 software was used in the flexible ligand docking simulations. Finally, PyMol software was used to represent these structures.

### 2.6 SCI model

All animal experiments were approved by the Intramural Animal Use and Care Committee of the Fourth Military Medical University and were conducted in accordance with institutional guidelines.

Adult female C57BL/6 mice (18–20 g, n=27) were randomly divided into three groups: (1) the uninjured group (9 mice); (2) the SCI + phosphate-buffered saline (PBS) group (9 mice); (3) the SCI + decitabine group (9 mice). Mice were first anesthetized with sodium pentobarbital, and then surgery for laminectomy was performed under a microscope at the level of the 10th thoracic vertebra. The T9-T10 segment of the spinal cord was fully exposed; subsequently, the mouse spinal cord was injured at the T9 segment using an arterial clamp for 1 minute, as previously reported (14). After clamp injury, the muscles and skin were closed. To restore spontaneous voiding, bladders were manually expressed twice daily for a week, and mice were treated with antibiotics (ceftriaxone 16 mg/kg) intraperitoneally twice a day for 5 days. In the drug-treated group, 15 μg/10 g decitabine (A10292, Adooq Bioscience, Nanjing, China) was administered intraperitoneally continuously for 7 days (once/day) postoperatively. The functional recovery of SCI mice was evaluated by 21-point Basso-Beattie-Bresnahan locomotion testing score (15). Treatment outcomes were evaluated once every 2 weeks (2w, 4w, 6w). At 6 weeks postoperative, motor evoked potentials (MEPs) of the hindlimb were assessed by electromyography. The samples of heart, kidney, liver, lung, stomach and spleen in SCI mice treated with decitabine for 6 weeks were stained with HE staining solution, and the HE staining characteristics were observed with an optical microscope.

### 2.7 Immunofluorescence staining and imaging and qRT-PCR

At 2 weeks post-injury, mice spinal cord tissues were fixed overnight in 4% paraformaldehyde. The samples were then permeabilized with 0.2% Triton X-100 (0.1%, Sigma-Aldrich) and blocked for 1h at 37°C in 1% BSA (6%, Bio-froxx, Germany) in 0.2% Triton X-100. After blocking, incubations were performed using specific primary antibodies (rabbit anti-CD206 [1:200, TD4149, Abmart], rabbit anti-iNOS [1:500, GB11119, Servicebio]) for 12 h followed by incubations with secondary antibodies for another 1 h. Images were captured using a fluorescence microscope (Olympus fluorescence microscope, Japan)

For quantitative real-time polymerase chain reaction, total mRNA was extracted from tissues (Life Technologies, USA) at 7dpi and 2 weeks post-injury using TRIzol-chloroform. cDNA was reversely transcribed with a PrimeScriptTM RT Master Mix (Takara, Dalian, China). PCR analysis was performed using a MiniOpticon Real-Time PCR system and Bio-Rad’s CFX manager software version 1.5. Finally, fold change was calculated as 2 to the -ΔΔCt power (2 -ΔΔCt). The qRT-PCR primers are summarized in **Table 1**.

**Table 1:** The primers for Real-Time Quantitative PCR.

Gene	Sequence
Vav1	TGTGAGAAGTTCGGCCTCAAG (Forward) CAGAGCAGACAGGGTGTAGAT (Reverse)
Itgb5	GAAGTGCCACCTCGTGTGAA (Forward) GGACCGTGGATTGCCAAAGT (Reverse)
B2m	GGCCCATCTTGCATTCTAGGG (Forward) CAGGCAACGGCTCTATATTGAAG (Reverse)
TNF-α	TCAGCCTCTTCTCATTCCTGC (Forward) TTGGTGGTTTGCTACGACGTG (Reverse)
IL1β	CTCCATGAGCTTTGTACAAGG (Forward)
	GGGGTTGACCATGTAGTCGT (Reverse)
IL-4	GGTCTCAACCCCCAGCTAGT (Forward)
	GCCGATGATCTCTCTCAAGTGAT (Reverse)
IL-10	CTTACTGACTGGCATGAGGATCA (Forward) GCAGCTCTAGGAGCATGTGG(Reverse)
TGF-β	TCTGCATTGCACTTATGCTGA (Forward)
	AAAGGGCGATCTAGTGATGGA (Reverse)
GAPDH	TTGTCTCCTGCGACTTCAAC (Forward)
	GTCATACCAGGAAATGAGCTTG (Reverse)
iNOS	GGGTCTTGTTAGCCTAGTCA (Forward)
	TGTTGTTGGGCTGGGAATAG (Reverse)
Arg-1	CTCCAAGCCAAAGTCCTTAGAG (Forward)
	AGGAGCTGTCATTAGGGACATC (Reverse)

### 2.8 Statistical analysis

Data were presented as the mean ± standard deviation, and all statistical analyses were performed with one-way analysis of variance using the GraphPad Prism software (GraphPad Prism Software, San Diego, California). Differences with p<0.05 were considered statistically significant.

## 3 Results

### 3.1 Identification of key regulatory genes

**Figure 2:**
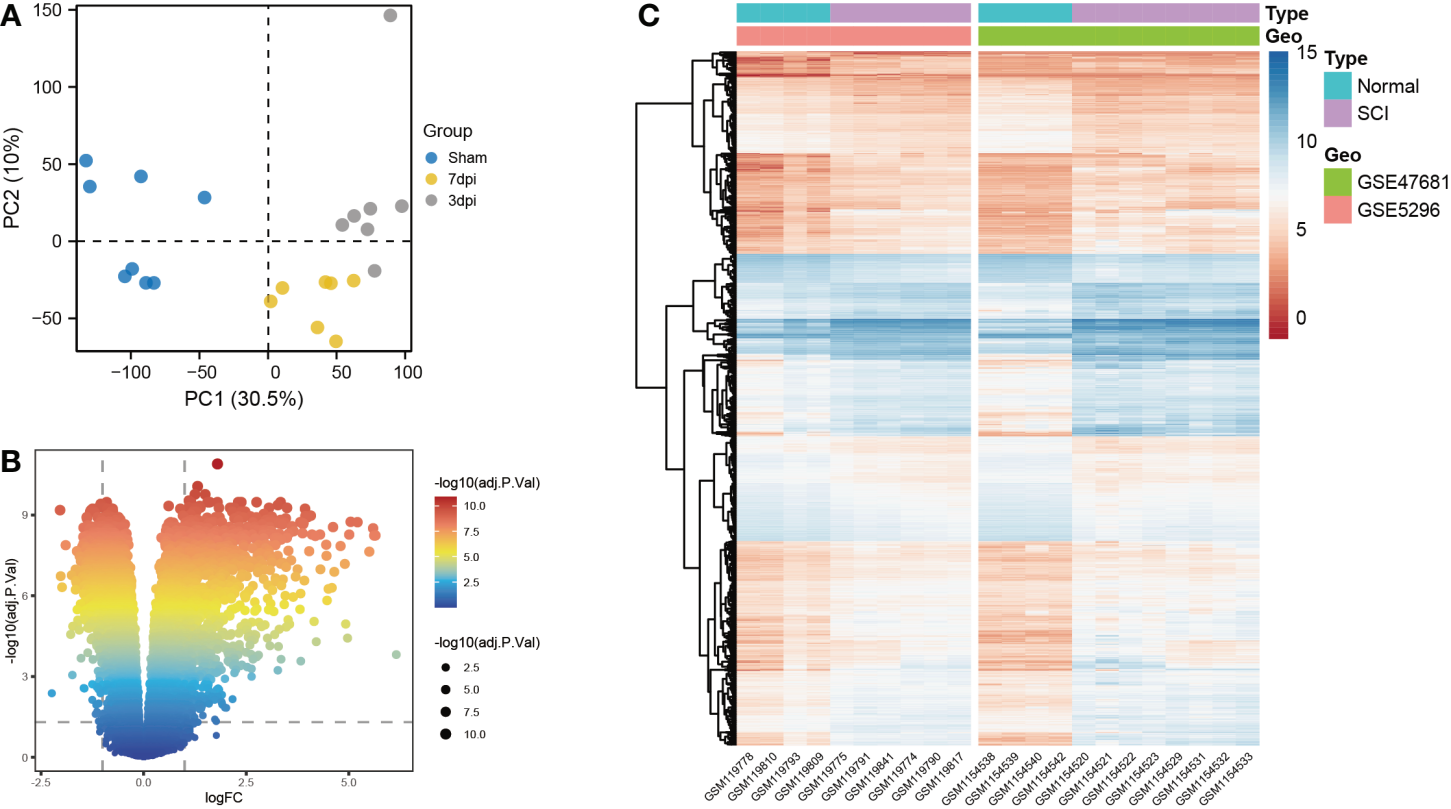
Screening for differentially expressed genes (DEGs) of bulk RNA-seq datasets. **(A)** Principal component analysis (PCA) analysis of GSE5296 and GSE47681. **(B)** Volcano plots of DEGs, screening criteria: |log 2 (fold-change) (FC)>1 and an adjusted p<0.05. **(C)** Heatmap of DEGs.

**Figure 3:**
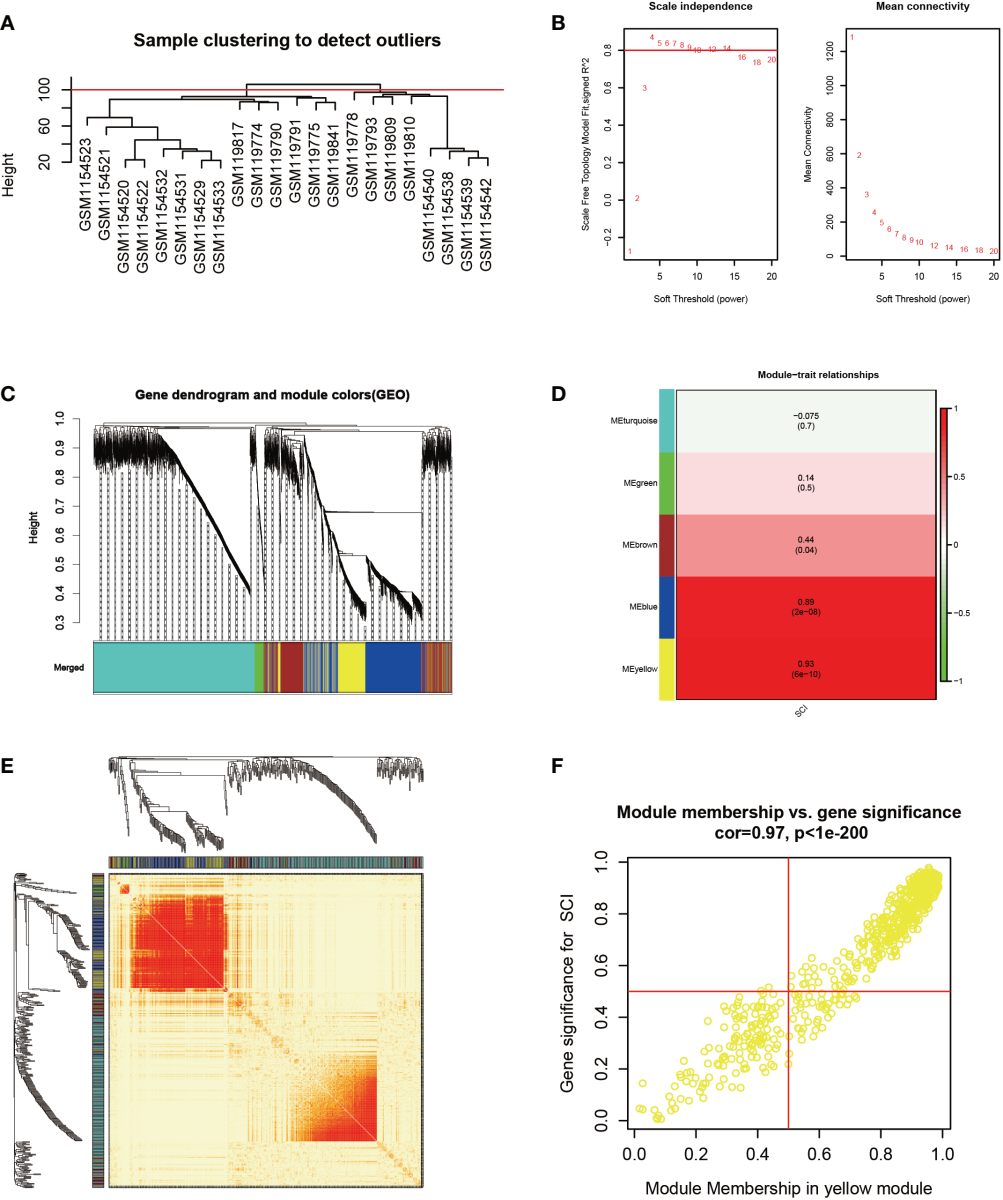
Identification of key modules genes of bulk RNA-seq datasets. **(A)** Outlier removal with “h > 100”. **(B)** Soft threshold setting according to R2 = 0.80. **(C)** The gene set is divided into 5 different modules. **(D)** The correlation of modules with SCI occurrence. **(E)** The heatmap of correlations between modules. **(F)** The correlation between yellow module and SCI gene significance. Gene significance for SCI refers to compare the correlation between a gene and its corresponding traits (absence of SCI).

Based on principal component analysis results of bulk RNA-seq datasets, the 22 sample characteristics of the SCI group (3 dpi and 7 dpi) were clearly distinguished from those of the sham group (**Figure 2A**). The heatmap and the volcano map indicated the presence of 1387 DEGs in these samples, including 947 upregulated genes and 440 downregulated genes (**Figures 2B, C**). All 22 samples in GSE5296 and GSE47681 met the condition of h <100 and were therefore included in the WGCNA analysis to search for key module genes of SCI (**Figure 3A**). In the analysis of these datasets, the smallest value (β=8) that resulted in a nearly scale-free network with a truncated scale-free fitting index of R2 = 0.80 (**Figure 3B**) was selected. Five modules with significant correlation with SCI were identified (**Figures 3C, D**), among which the yellow module was the most significant (module feature correlation=0.93). The heatmap of correlations between modules is shown in **Figure 3E**. Finally, the correlation coefficient between the 686 genes in the yellow module and the importance of SCI genes was 0.97 (**Figure 3F**).

### 3.2 Comprehensive analysis of key regulatory genes

**Figure 4:**
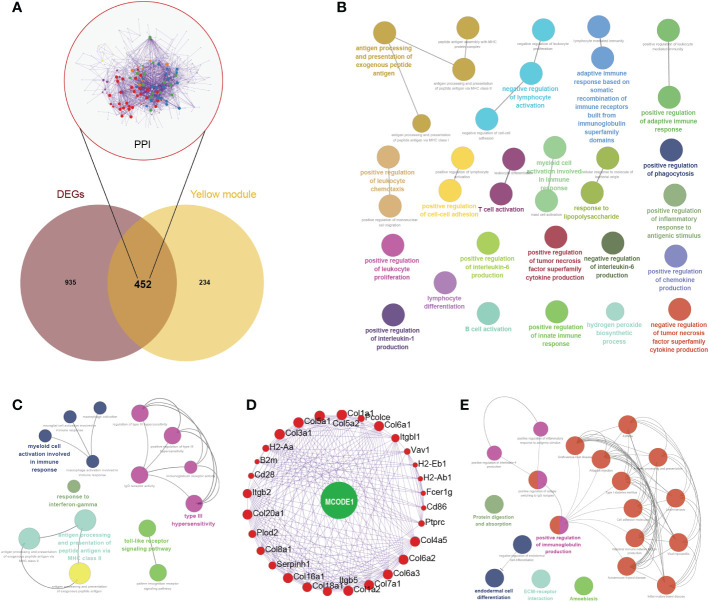
Comprehensive analysis of key regulatory genes. **(A)** Venn diagram of intersected genes between differentially expressed genes (DEGs) and yellow module genes. Protein-protein interaction network of 452 key regulatory genes were also displayed. **(B)** Biological pathways of these 452 key regulatory genes. **(C)** Immune pathway of key regulatory genes. **(D)** Hub genes were selected in the protein–protein interaction through the MCODE application. **(E)** Biological pathways of these 29 hub genes.

In the Venn diagram, 452 genes were intersected between DEGs and yellow module genes (**Figure 4A**). Functional enrichment showed that these regulatory genes were mainly enriched in biological pathways such as negative regulation of lymphocyte activation, antigen processing and presentation of exogenous peptide antigen, and positive regulation of inflammatory response to antigenic stimulus (**Figure 4B**). Further immune pathway enrichment analysis showed that key regulatory genes involved in response to interferon-gamma, toll-like receptor signaling pathway (**Figure 4C**). Overall, 29 hub genes were selected in the protein–protein interaction through the MCODE application (**Figures 4A, D**). These hub genes were involved in the positive regulation of immunoglobulin production, ECM-receptor, endodermal cell differentiation (**Figures 4E**).

### 3.3 Analysis of immune cell infiltration

**Figure 5:**
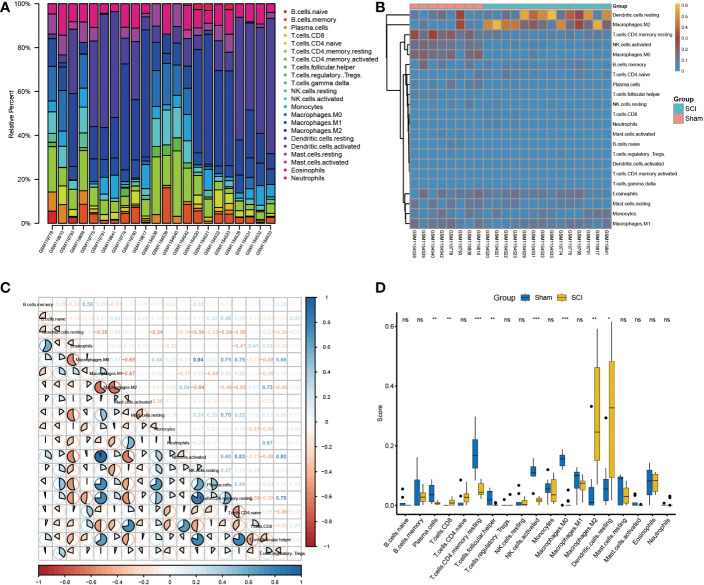
Immune infiltration analysis of bulk RNA-seq datasets. **(A)** Immune infiltration of the 22 immune cells in GSE5296 and GSE47681dataset. **(B)**Immune infiltration heatmap in GSE5296 and GSE47681dataset. **(C)** Correlation heatmap of immune cells. **(D)** The expression difference of 22 immune cells between SCI and uninjured samples. *P < 0.05, **P < 0.01, ***P < 0.001.

Considering the important role of changes in the immune microenvironment on post-SCI regeneration and the enrichment of key regulatory genes in immune-related pathways, we calculated the contents of 22 immune cells in the samples based on the CIBERSORT algorithm (**Figure 5A**). The heatmap of immune cell abundance in each sample is shown in **Figure 5B**. In addition, the correlation of immune cells in these samples was also analyzed (**Figure 5C**). Among them, M0 macrophages had the strongest positive correlation with activated NK cells (r=0.94), while M1 macrophages had the strongest negative correlation with M2 macrophages (r=-0.67). In addition, the levels of CD8^+^ T cells, M2 macrophages, and resting dendritic cells were higher in the SCI group than in the sham group. Meanwhile, the levels of plasma cells, memory CD4^+^ T cells, T follicular helper cells, activated NK cells, and M0 macrophages were lower in the SCI group than in the sham group (**Figure 5D**).

**Figure 6:**
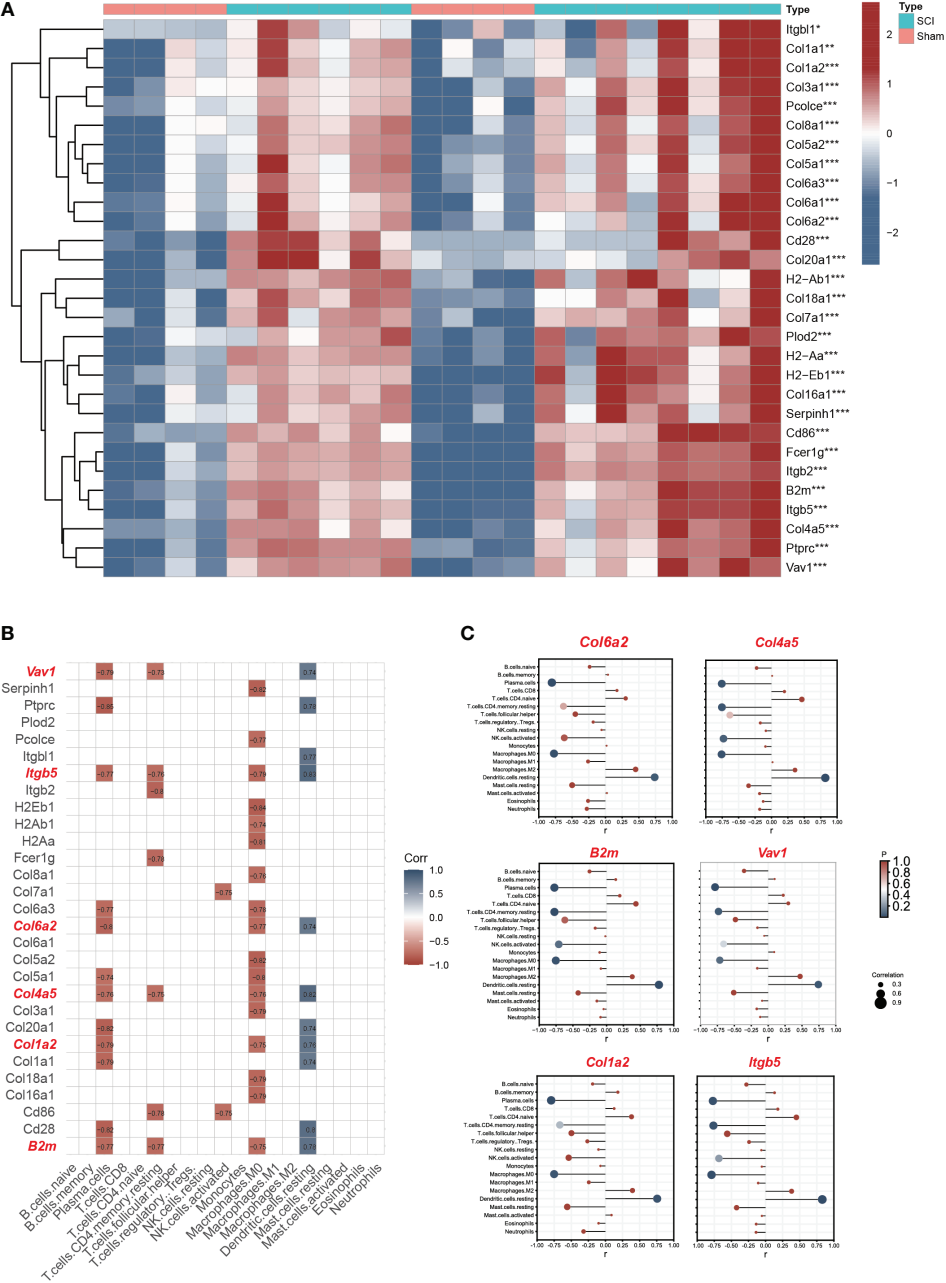
Screening immune hub genes based on bulk RNA seq datasets. **(A)** The expression matrix heatmap of 29 hub genes. **(B)**The correlation analyses between hub genes and immune characteristics between SCI and uninjured samples. The criteria for selection of key immune genes related to SCI were as follows: >2 immune cell correlation, correlation coefficient >0.3, and p<0.05. **(C)** The lollipop plot of six immune hub genes (*Vav1, Itgb5, B2m, Col1a2, Col4a5, Col6a2*). *P < 0.05, **P < 0.01, ***P < 0.001.

The expression matrix heatmap of 29 hub genes is shown in **Figure 6A**. Based on the screening criteria, six genes (*Vav1, Itgb5, B2m, Col1a2, Col4a5, Col6a2*) were identified as immune hub genes (**Figure 6B**). According to the results of lollipop plot (**Figure 6C**), *Itgb5* had the strongest positive correlation with resting dendritic cells (r=0.83), and *Col6a2* had the strongest negative correlation with plasma cells (r=-0.80).

### 3.4 ScRNA-seq analysis

After integrating data from the sham group (10,227 cells), the 3-dpi group (12,783 cells), and the 7-dpi group (16,447 cells) in the GSE189070 dataset, a total of 39,457 cells were identified. Low-quality cells were filtered using an nFeature_RNA cutoff,400; nCount_RNA cutoff, 1000; and percent mitochondrial genes cutoff, 15%. Subsequently, 34,331 high-quality cells from the sham group (7,499 cells), the 3-dpi group (11,496 cells), and the 7-dpi group (15,336 cells) were screened for downstream analysis. After selecting the top 2000 highly variable genes, linear dimensionality reduction was performed to identify the available dimensions of the dataset. First 20 principal components were used to generate the UMAP visualization. Finally, 10 cell clusters were identified, and UMAP plots were visualized between different time points and different groups of cell clusters.

**Figure 7:**
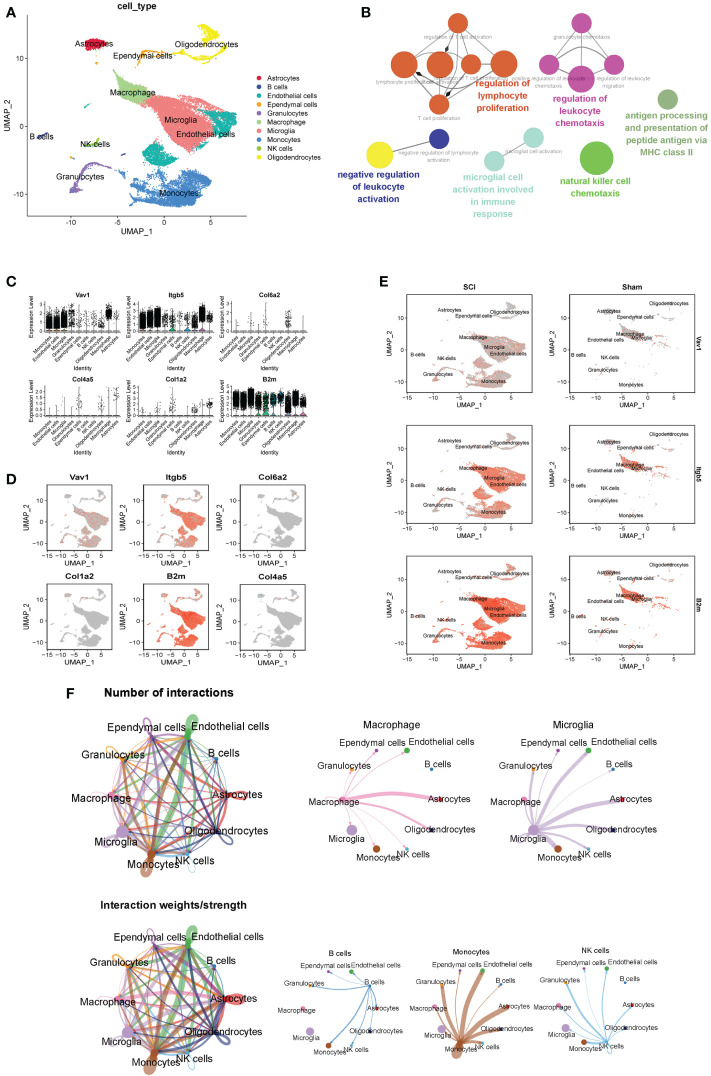
ScRNA-seq analysis of the subacute phase of spinal cord injury. **(A)** Nine cell clusters (astrocytes, B cells, endothelial cells, ependymal cells, granulocytes, microglia, monocytes, NK cells, oligodendrocytes) were identified on the UMAP plot. **(B)** The functional enrichment analysis of differentially expressed genes in different cell clusters. **(C)** The violin plots of six immune hub genes in each cluster. **(D)** The UMAP plots of six immune hub genes in each cluster. **(E)** Among these immune hub genes, *Vav1* (Log FC=0.37), *Itgb5* (Log FC=0.75), and *B2m* (Log FC=0.75) are characteristic genes of microglia/macrophages. **(F)** The number and strength of interactions between 10 cell clusters.

Ten cell clusters (astrocytes, B cells, endothelial cells, ependymal cells, granulocytes, microglia, macrophages, monocytes, NK cells, oligodendrocytes) were identified on the UMAP plot (**Figure 7A**). Neurons were not annotated because of their susceptibility to death during enzymatic dissociation. The results of functional enrichment analysis showed that the functions of DEGs of cell clusters were mainly enriched in regulation of lymphocyte proliferation, negative regulation of leukocyte activation, and microglial cell activation in immune pathway. (**Figure 7B**). The percentage of expression of immune hub genes in each cluster are is as violin plots and UMAP plots (**Figures 7C, D**). Among these immune hub genes, *Vav1* (Log FC=0.37), *Itgb5* (Log FC=0.75), and *B2m* (Log FC=0.75) are characteristic genes of microglia/macrophages (**Figure 7E**). We also found that *B2m* and *Itgb5* were mainly located in microglia (*Trem2*, *C1qa*, *Fcrls*), while *Vav1* was mainly located in macrophages (*Cx3cr1*, *Csf1r*, *Cst3*). The number and strength of interactions between 10 cell clusters are shown in **Figure 7F**. For immune cells, B cells were strongly associated with monocytes, macrophages with astrocytes, monocytes with endothelial cells, and NK cells with ganglion cells. It was worth noting that microglia have strong correlation with endothelial cells, astrocytes and oligodendrocytes.

### 3.5 Validation of immune hub genes and drug prediction

**Figure 8:**
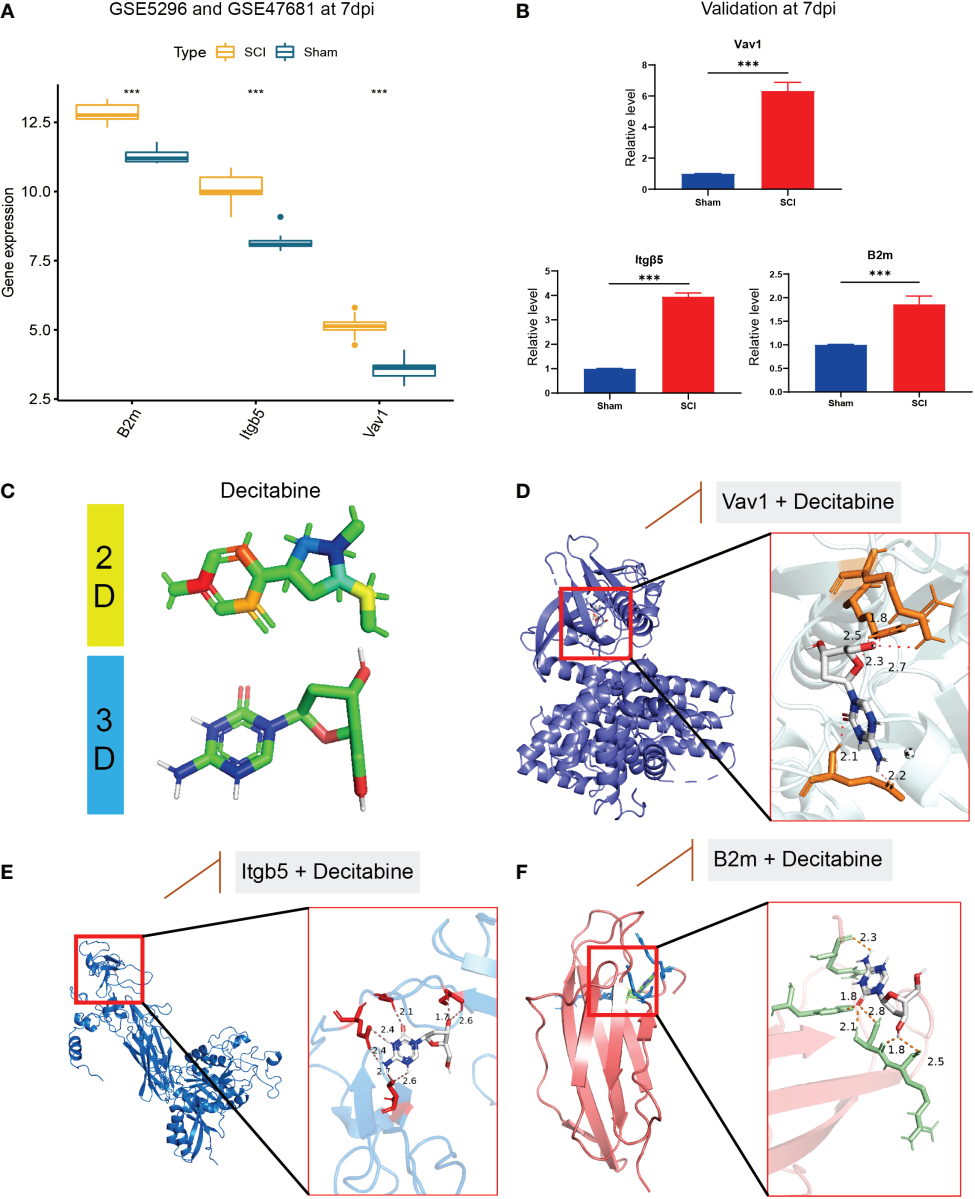
Validation of immune hub genes and drug prediction. **(A)** In the bulk RNA-seq datasets, *B2m, Itgb5, and Vav1* were all highly expressed in the SCI group, **(B)** consistent with the validation results of qRT-PCR in 7 dpi. **(C)** The two-dimensional and three-dimensional structure of decitabine. **(D–F)** the possible binding sites of decitabine with *B2m, Itgb5*, and *Vav1* proteins. &Statistically significant difference appeared on this day. ***P < 0.001.

In the bulk RNA-seq datasets, *B2m, Itgb5, and Vav1* were all highly expressed in the SCI group (**Figure 8A**), consistent with the validation results of qRT-PCR in 7 dpi (**Figure 8B**). Based on the DSigDB database results, we speculate that decitabine (P=0.02) may be a small molecule compound that binds to these immune hub genes (**Figure 8C**). Finally, the possible binding sites of decitabine with *B2m, Itgb5*, and *Vav1* proteins were successfully predicted using AutoDock software (**Figures 8D–F**).

### 3.6 *In vivo* functional and histological assessment

**Figure 9:**
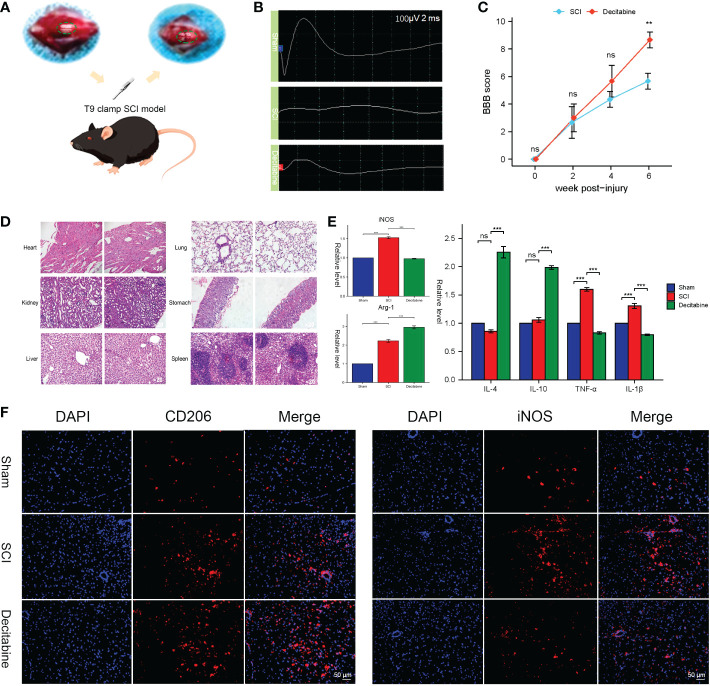
*In vivo* functional and histological assessment. **(A)** The spinal cord injury model of T9 by arterial clamp. **(B)** Electromyography was observed at 6 weeks post-SCI in uninjured group, the SCI + phosphate-buffered saline (PBS) group and the SCI + decitabine group. **(C)** The Basso-Beattie-Bresnahan locomotion testing score at 6 weeks post-SCI in the SCI + PBS group and the SCI + decitabine group (*n* = 6 in each group). **(D)** HE-stained sections of the heart, liver, spleen, lung and kidney of the mice at 6 weeks post-SCI in the SCI + decitabine group (magnification, ×200). **(E)** The expression level of iNOS, Arg, IL-4, IL-10, TGF-α, and IL-1β at 2 weeks in uninjured group, the SCI + PBS group and the SCI + decitabine group. **(F)**The immunofluorescence staining of CD206 and iNOS at 2 weeks post-SCI. &Statistically significant difference appeared on this day. **P < 0.01, ***P < 0.001.

After exposing the spinal cord of the T9 segment, we successfully made a T9 clamp SCI model (**Figure 9A**). Improved MEPs were observed at 6 weeks post-SCI in the decitabine group (**Figure 9B**). In addition, the Basso-Beattie-Bresnahan locomotion testing score was also improved 6 weeks post-SCI in the decitabine group, although the improvement in the decitabine group was not statistically significant in the first 4 weeks post-SCI (**Figure 9C**). Notably, HE staining of the heart, kidney, liver, lung, stomach and spleen did not show significant morphological changes in SCI mice treated with decitabine for 6 weeks (**Figure 9D**).Follow-up analysis of the tissue samples from each group at 2 weeks post-SCI showed that compared with the SCI + PBS group, the decitabine group had higher levels of Arg-1, IL-4, and IL-10 (P<0.05) (**Figure 9E**). Meanwhile, the decitabine group showed lower levels of iNOS, TGF-α and IL-1β (P<0.05). Finally, under decitabine stimulation, CD206 showed stronger red fluorescence, while iNOS showed weak red fluorescence. All in all, these results all support the transition of macrophages/microglia from M1 type to M2 type (**Figure 9F**).

## 4 Discussion

SCI often leads to irreversible neurological impairment and also has a significant socioeconomic impact on both the patients and the healthcare system (16). The altered immune microenvironment after SCI hinders neural regeneration and repair, but related studies have been met with conflicting findings (17). Next-generation sequencing is a novel modality that enabled the discovery of the altered immune microenvironment after SCI (18). The current study was based on Bulk RNA-seq and scRNA-seq and explored the immune hub genes and immune cell composition in the subacute phase of SCI. Drugs to promote regeneration by regulating the polarization status of microglia were successfully identified. Importantly, the study shows that regulating the dual role of the immune microenvironment is a potential strategy in managing neuroinflammation during SCI.

Various immune cells, including neutrophils, macrophages, microglia, B lymphocytes, and T lymphocytes, have been shown to be involved in the inflammatory response after SCI (19). Concurrently, the composition and phenotype of immune cells are also altered with the stage of injury and the signals in the injury microenvironment. For example, microglia/macrophages, T cells, and B cells are capable of both pro-inflammatory and anti-inflammatory responses in the injured spinal cord, thereby regulating secondary injury after SCI. Microglia/macrophages start to accumulate at 3 days after SCI and peak at 7 dpi (20). Consistently, immune infiltration analysis in the current study showed that levels of M2 macrophages were higher in the SCI group than in the sham group. Interestingly, we also found changes in the immune microenvironment of some acquired immune cells (resting dendritic cells, plasma cells and activated NK cell), which is closely related to the destruction of the blood–spinal cord barrier.

By combining the screening of DEGs, WGCNA, and immune infiltration analysis, *B2m, Itgb5, and Vav1*, were identified as immune hub genes of SCI. Cartarozzi et al. reported that *B2m* knockout could lead to weakened neuronal axon regeneration after SCI by enhancing microglia activation (21). Another reptile-based study also showed that *Vav1* can regulate the inflammatory reaction mediated by the macrophage migration inhibitory factor to promote the regeneration of the nervous system (22). Although the immune invasion of *Itgb5* in gastric cancer and glioblastoma has been well demonstrated, its role in SCI still needs further research (23, 24).

Activated microglia and blood-infiltrating macrophages are indistinguishable after SCI, and both exacerbate inflammatory injury by releasing protein hydrolases, reactive oxygen species, and inflammatory factors (25). However, the heterogeneity of the microglia and macrophages prevents the in-depth study of post-SCI regeneration, which can be further identified by scRNA-seq. Wang et al. performed scRNA-seq on immune cells sorted by flow cytometry after SCI and divided microglia and macrophages into three clusters and seven clusters, respectively (18). Highly accurate differentiation of microglia identified that targeting Hif1α may contribute to axonal regeneration and functional recovery after SCI. Another scRNA-seq study showed the dynamic changes and molecular characteristics of microglia within 38 days after SCI, providing additional evidence for strategies targeting microglia to promote neurological function repair (26). The current study identified the clusters of macrophages (*Cx3cr1*, *Csf1r*, *Cst3*) and microglia (*Trem2*, *C1qa*, *Fcrls*) and found that *B2m* and *Itgb5* were mainly located in microglia, while *Vav1* was mainly located in macrophages.

Drug discovery is the process of identifying potential new drugs from existing molecular databases and is a necessary part of the human pathway to treating disease. There are two key components involved in drug discovery: the protein (target) and the small molecule (ligand), which also known as protein-ligand interaction. In the present study, decitabine was shown to act as a ligand to inhibit the activity of B2m, Itgb5, and Vav1 proteins. Among them, decitabine exerts its pharmacological effects mainly through polar covalent bonds with GLU-524, ARG-54 and ARG550 of the VAV1 protein, with SER-534, CYS-489, GLY-492, GLU-502 of the Itgb5 protein and with ARG-12, ARG-97 and TRP-95 of the Itgb5 protein. Of course, the simulated docking results need further validation by liquid chromatography and mass spectrometry.

Whether neurons and axons can regenerate after SCI depend not only on their original growth ability, but also on the neurovascular microenvironment and epigenetic changes, which is mainly embodied in DNA methylation and histone acetylation (27). Among these, DNA methylation affects many aspects of neural stem cell maintenance and proliferation, neuronal differentiation and maturation, and synaptogenesis (28). Decitabine is a demethylating drug that can inhibit DNA methyltransferase, reduce DNA methylation, and inhibit tumor cell proliferation, and it has been the primary drug for elderly AML patients (29). Decitabine can also reduce the proliferation and vitality of tumor cells in malignant meningioma and glioblastoma (30, 31). Interestingly, a recent study showed that decitabine can improve white matter lesions caused by chronic cerebral hypoperfusion (32). To our best knowledge, this study is the first to report that decitabine may promote the recovery of neural function after SCI by promoting the transformation of proinflammatory macrophages/microglia to anti-inflammatory macrophages/microglia.

However, this study also has some limitations. First, in the Bulk RNA-seq analyses, although data were preprocessed using the “sva” package, batch effect of different datasets should also be addressed. Meanwhile, deconvolution of scRNA-seq to further discover the consistency of immune cells with bulk RNA seq is our follow-up research work. Second, although no obvious side effects were observed within 6 weeks, the mice injected with small doses of decitabine died in succession at 8 weeks. Therefore, the injection dose and safety of decitabine need to be further evaluated in *in vivo* and *in vitro* experiments. Thirdly, restricted by the fact that Autodock is limited to the docking of a single receptor ligand, there was a possibility that decitabine may bind to genes other than the hub gene in macrophages/microglia for pharmacological effects. Lastly, hub gene knockdown to clarify the role of hub genes (B2m, Itgb5, and Vav1) in SCI was a topic that we will explore in the future.

In conclusion, the current study identified the changes in immune cell clusters and immune hub genes after SCI using a combination of Bulk RNA-seq and scRNA-seq and discovered the drugs that can regulate macrophage/microglia polarization to improve neural function. The exploration of the immune microenvironment after SCI and the identification of immune hub genes may provide additional scientific evidence for developing more effective treatment modalities targeting the microglia in SCI.

## Data availability statement

The original contributions presented in the study are included in the article/Supplementary Material. Further inquiries can be directed to the corresponding authors.

## Ethics statement

The animal study was reviewed and approved by the Intramural Animal Use and Care Committee of the Fourth Military Medical University.

## Author contributions

QZ, BY and YZ: manuscript preparation, data analysis, and the research conception. YT and SY: Animal experiment and qRT-PCR. YC and HW: manuscript revision. All authors contributed to the article and approved the submitted version.

## Footnotes

1
http://www.ncbi.nlm.nih.gov/geo/

2
http://metascape.org

3
https://cn.string-db.org

4
https://cibersort.stanford.edu

5
http://dsigdb.tan-lab.org/DSigDBv1.0/

6
https://pubchem.ncbi.nlm.nih.gov/

7
https://www.pdbus.org/
